# 6-Phenyl­benzo[*d*]naphtho­[2,3-*b*]thio­phene

**DOI:** 10.1107/S1600536812049471

**Published:** 2012-12-08

**Authors:** V. Silambarasan, T. Srinivasan, R. Sivasakthikumaran, A. K. Mohanakrishnan, D. Velmurugan

**Affiliations:** a CAS in Crystallography and Biophysics, University of Madras, Guindy Campus, Chennai-25, India; b Department of Organic Chemistry, University of Madras, Guindy Campus, Chennai-25, India

## Abstract

In the title compound, C_22_H_14_S, the r.m.s. deviation from the mean plane of the four-fused-ring naphtho­thio­phene unit is 0.056 Å. The dihedral angle between the naphtho­thio­phene plane and the pendant phenyl ring is 67.24 (6)°. In the crystal, weak C—H⋯π and π–π stacking [minimum centroid–centroid separation = 3.7466 (10) Å] inter­actions are observed, which together lead to (010) sheets.

## Related literature

For background to the biological activity of benzothio­phene derivatives, see: Isloora *et al.* (2010 ▶).
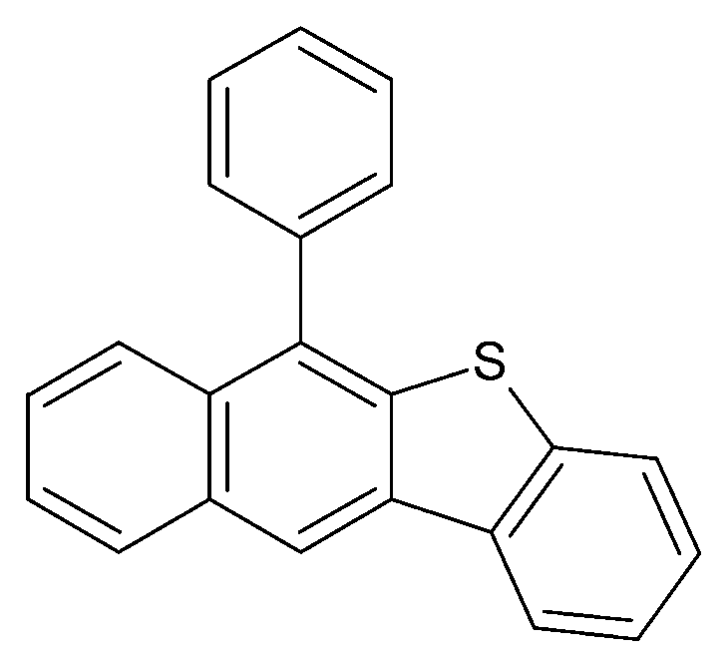


## Experimental

### 

#### Crystal data

C_22_H_14_S
*M* *_r_* = 310.40
Orthorhombic, 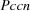

*a* = 12.6752 (10) Å
*b* = 28.578 (2) Å
*c* = 8.5659 (6) Å
*V* = 3102.8 (4) Å^3^
*Z* = 8
Mo *K*α radiation
μ = 0.21 mm^−1^
*T* = 293 K
0.20 × 0.20 × 0.20 mm

#### Data collection

Bruker APEXII CCD diffractometer
16518 measured reflections
3855 independent reflections
2892 reflections with *I* > 2σ(*I*)
*R*_int_ = 0.031

#### Refinement

*R*[*F* ^2^ > 2σ(*F* ^2^)] = 0.043
*wR*(*F* ^2^) = 0.118
*S* = 1.03
3855 reflections
208 parameters
H-atom parameters constrained
Δρ_max_ = 0.21 e Å^−3^
Δρ_min_ = −0.24 e Å^−3^

### 

Data collection: *APEX2* (Bruker, 2008 ▶); cell refinement: *SAINT* (Bruker, 2008 ▶); data reduction: *SAINT*; program(s) used to solve structure: *SHELXS97* (Sheldrick, 2008 ▶); program(s) used to refine structure: *SHELXL97* (Sheldrick, 2008 ▶); molecular graphics: *ORTEP-3* (Farrugia, 2012) ▶; software used to prepare material for publication: *SHELXL97* and *PLATON* (Spek, 2009 ▶).

## Supplementary Material

Crystal structure: contains datablock(s) global, I. DOI: 10.1107/S1600536812049471/hb6995sup1.cif

Structure factors: contains datablock(s) I. DOI: 10.1107/S1600536812049471/hb6995Isup2.hkl

Supplementary material file. DOI: 10.1107/S1600536812049471/hb6995Isup3.cml

Additional supplementary materials:  crystallographic information; 3D view; checkCIF report

## Figures and Tables

**Table 1:** Hydrogen-bond geometry (Å, °) *Cg*2 and *Cg*3 are the centroids of the C1-C6 and C10–C16 rings, respectively.

*D*—H⋯*A*	*D*—H	H⋯*A*	*D*⋯*A*	*D*—H⋯*A*
C5—H5⋯*Cg*2^i^	0.93	2.94	3.8138 (19)	158
C13—H13⋯*Cg*3^i^	0.93	2.64	3.5399 (17)	163

Symmetry code: (i) 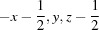.

**Fig. 1.**
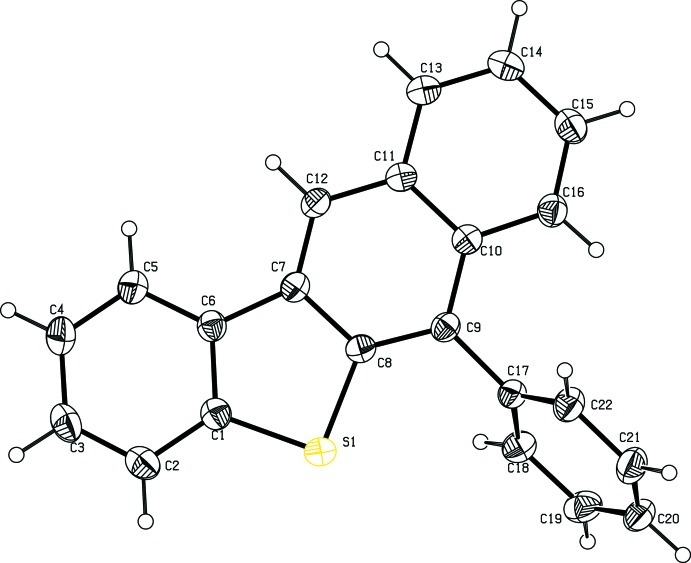
The molecular structure of the title compound, showing displacement ellipsoids drawn at the 30% probability level.

**Fig. 2.**
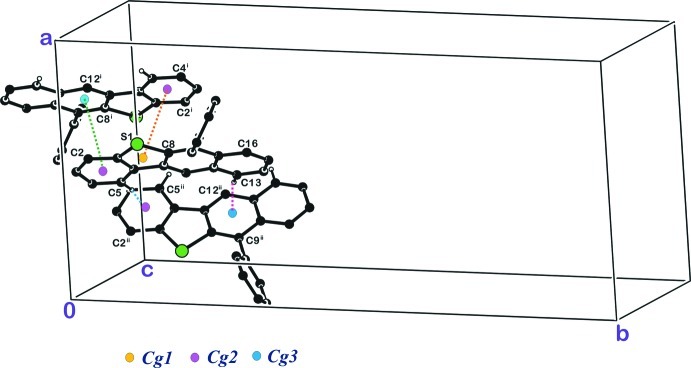
The π···π and C—H···π interactions (dotted lines) in the title compound. Cg1, Cg2 and Cg3 are the centroids of the S1/C1/C6/C7/C8, C1—C6, C7—C12 rings. [Symmetry code: (i) 1 - x,-y,2 - z; (ii) 1/2 - x,y,1/2 + z].

## supplementary crystallographic information

### Comment

Some benzo[*b*]thiophene derivatives show significant antimicrobial and anti-inflammatory activities (Isloora *et al.*, 2010). As part of our own studies in this area, an X-ray study of the title compound was carried out.

Fig. 1 shows the *ORTEP* representation of the molecular structure of the title compound with atoms at the 30% probability level. The naphthothiophene moiety is almost planar with an r.m.s. deviation of fitted atoms = -0.0065 (1)°. The dihedral angle between the naphthothiophene plane and the attached benzene [C17—C22] ring is 67.24 (6)°. The thiophene ring is almost planar, with maximum deviation of 0.014 (1) Å.

In the crystal, C—H···π interactions occur (Table 1).

### Experimental

The benzo[*b*]thiophen-3-yl(2-(phenyl(pivaloyloxy)methyl)phenyl) methyl pivalate (0.73 g, 1.60 mmol) upon interaction with ZnBr2 (0.02 g, 0.13 mmol) followed by removal of solvent and column chromatographic purification (silica gel; hexane-ethyl acetate, 99:1) gave the compound as a colorless solid (0.50 g, 72%). Colourless blocks were obtained by slow evaporation of a solution of the title compound in acetone at room temperature.

### Refinement

All H atoms were fixed geometrically and allowed to ride on their parent C atoms, with C—H distances fixed in the range 0.93–0.97 Å with *U*_iso_(H) = 1.5*U*_eq_(C) for methyl H 1.2*U*_eq_(C) for other H atoms.

### Crystal data

**Table:**

C_22_H_14_S	*F*(000) = 1296
*M**_r_* = 310.40	*D*_x_ = 1.329 Mg m^−^^3^
Orthorhombic, *P**c**c**n*	Mo *K*α radiation, λ = 0.71073 Å
Hall symbol: -P 2ab 2ac	Cell parameters from 3855 reflections
*a* = 12.6752 (10) Å	θ = 1.8–28.4°
*b* = 28.578 (2) Å	µ = 0.21 mm^−^^1^
*c* = 8.5659 (6) Å	*T* = 293 K
*V* = 3102.8 (4) Å^3^	Block, colourless
*Z* = 8	0.20 × 0.20 × 0.20 mm

### Data collection

**Table:**

Bruker APEXII CCD diffractometer	2892 reflections with *I* > 2σ(*I*)
Radiation source: fine-focus sealed tube	*R*_int_ = 0.031
Graphite monochromator	θ_max_ = 28.4°, θ_min_ = 1.8°
ω and φ scans	*h* = −16→16
16518 measured reflections	*k* = −34→38
3855 independent reflections	*l* = −9→11

### Refinement

**Table:**

Refinement on *F*^2^	Primary atom site location: structure-invariant direct methods
Least-squares matrix: full	Secondary atom site location: difference Fourier map
*R*[*F*^2^ > 2σ(*F*^2^)] = 0.043	Hydrogen site location: inferred from neighbouring sites
*wR*(*F*^2^) = 0.118	H-atom parameters constrained
*S* = 1.03	*w* = 1/[σ^2^(*F*_o_^2^) + (0.0571*P*)^2^ + 0.6864*P*] where *P* = (*F*_o_^2^ + 2*F*_c_^2^)/3
3855 reflections	(Δ/σ)_max_ < 0.001
208 parameters	Δρ_max_ = 0.21 e Å^−^^3^
0 restraints	Δρ_min_ = −0.24 e Å^−^^3^

### Special details

**Table:**

Geometry. All e.s.d.'s (except the e.s.d. in the dihedral angle between two l.s. planes) are estimated using the full covariance matrix. The cell e.s.d.'s are taken into account individually in the estimation of e.s.d.'s in distances, angles and torsion angles; correlations between e.s.d.'s in cell parameters are only used when they are defined by crystal symmetry. An approximate (isotropic) treatment of cell e.s.d.'s is used for estimating e.s.d.'s involving l.s. planes.
Refinement. Refinement of *F*^2^ against ALL reflections. The weighted *R*-factor *wR* and goodness of fit *S* are based on *F*^2^, conventional *R*-factors *R* are based on *F*, with *F* set to zero for negative *F*^2^. The threshold expression of *F*^2^ > σ(*F*^2^) is used only for calculating *R*-factors(gt) *etc*. and is not relevant to the choice of reflections for refinement. *R*-factors based on *F*^2^ are statistically about twice as large as those based on *F*, and *R*- factors based on ALL data will be even larger.

### Fractional atomic coordinates and isotropic or equivalent isotropic displacement parameters (Å^2^)

**Table:**

	*x*	*y*	*z*	*U*_iso_*/*U*_eq_	
S1	0.49436 (3)	0.039709 (15)	0.71196 (5)	0.04701 (14)	
C11	0.34912 (11)	0.12197 (5)	1.10829 (17)	0.0367 (3)	
C10	0.41403 (11)	0.14729 (5)	1.00101 (17)	0.0361 (3)	
C17	0.52550 (12)	0.14890 (5)	0.75491 (18)	0.0375 (3)	
C7	0.37407 (11)	0.05119 (5)	0.95979 (18)	0.0376 (3)	
C9	0.46095 (11)	0.12338 (5)	0.87264 (17)	0.0361 (3)	
C13	0.30387 (12)	0.14611 (6)	1.23609 (19)	0.0444 (4)	
H13	0.2633	0.1297	1.3080	0.053*	
C8	0.44145 (11)	0.07613 (5)	0.85662 (17)	0.0369 (3)	
C22	0.62230 (13)	0.16834 (6)	0.7927 (2)	0.0471 (4)	
H22	0.6492	0.1648	0.8930	0.057*	
C1	0.42526 (12)	−0.00859 (5)	0.78516 (19)	0.0411 (3)	
C6	0.36343 (12)	0.00252 (5)	0.91445 (18)	0.0388 (3)	
C12	0.32950 (12)	0.07416 (5)	1.08341 (19)	0.0397 (3)	
H12	0.2857	0.0579	1.1517	0.048*	
C16	0.42662 (13)	0.19611 (6)	1.0261 (2)	0.0437 (4)	
H16	0.4677	0.2134	0.9572	0.052*	
C18	0.48824 (13)	0.15417 (6)	0.6039 (2)	0.0457 (4)	
H18	0.4244	0.1406	0.5755	0.055*	
C19	0.54498 (15)	0.17947 (7)	0.4950 (2)	0.0533 (4)	
H19	0.5189	0.1832	0.3943	0.064*	
C5	0.30009 (14)	−0.03191 (6)	0.9809 (2)	0.0491 (4)	
H5	0.2582	−0.0250	1.0670	0.059*	
C20	0.63982 (15)	0.19908 (6)	0.5357 (2)	0.0528 (4)	
H20	0.6774	0.2166	0.4629	0.063*	
C15	0.37981 (14)	0.21805 (6)	1.1489 (2)	0.0499 (4)	
H15	0.3886	0.2501	1.1620	0.060*	
C2	0.42771 (15)	−0.05383 (6)	0.7253 (2)	0.0493 (4)	
H2	0.4712	−0.0614	0.6415	0.059*	
C21	0.67926 (14)	0.19303 (6)	0.6823 (2)	0.0536 (4)	
H21	0.7447	0.2056	0.7083	0.064*	
C3	0.36430 (15)	−0.08691 (6)	0.7931 (2)	0.0539 (5)	
H3	0.3645	−0.1173	0.7539	0.065*	
C4	0.29989 (15)	−0.07614 (6)	0.9188 (2)	0.0554 (5)	
H4	0.2563	−0.0990	0.9613	0.066*	
C14	0.31846 (14)	0.19275 (6)	1.2558 (2)	0.0501 (4)	
H14	0.2877	0.2080	1.3403	0.060*	

### Atomic displacement parameters (Å^2^)

**Table:**

	*U*^11^	*U*^22^	*U*^33^	*U*^12^	*U*^13^	*U*^23^
S1	0.0557 (3)	0.0449 (2)	0.0404 (3)	−0.00271 (18)	0.01194 (18)	−0.00246 (18)
C11	0.0346 (7)	0.0416 (8)	0.0341 (8)	0.0018 (6)	−0.0010 (6)	0.0011 (6)
C10	0.0347 (7)	0.0395 (8)	0.0340 (8)	0.0009 (6)	−0.0037 (6)	0.0022 (6)
C17	0.0415 (8)	0.0346 (8)	0.0365 (8)	0.0008 (6)	0.0039 (6)	0.0026 (6)
C7	0.0372 (7)	0.0384 (8)	0.0371 (8)	−0.0013 (6)	−0.0001 (6)	0.0039 (6)
C9	0.0355 (7)	0.0395 (8)	0.0333 (8)	−0.0024 (6)	−0.0016 (6)	0.0037 (6)
C13	0.0418 (8)	0.0510 (10)	0.0404 (9)	0.0017 (7)	0.0051 (7)	−0.0001 (7)
C8	0.0382 (7)	0.0394 (8)	0.0332 (8)	0.0001 (6)	0.0013 (6)	0.0010 (6)
C22	0.0508 (9)	0.0483 (9)	0.0423 (9)	−0.0087 (7)	−0.0026 (7)	0.0066 (8)
C1	0.0437 (8)	0.0392 (8)	0.0403 (9)	0.0014 (6)	−0.0038 (7)	0.0014 (7)
C6	0.0416 (8)	0.0356 (8)	0.0391 (8)	0.0010 (6)	−0.0025 (6)	0.0028 (6)
C12	0.0392 (7)	0.0410 (8)	0.0390 (8)	−0.0020 (6)	0.0050 (6)	0.0055 (7)
C16	0.0485 (8)	0.0392 (8)	0.0435 (9)	−0.0025 (7)	−0.0016 (7)	0.0033 (7)
C18	0.0454 (8)	0.0521 (10)	0.0397 (9)	−0.0004 (7)	0.0008 (7)	0.0042 (7)
C19	0.0608 (10)	0.0604 (11)	0.0386 (9)	0.0084 (9)	0.0052 (8)	0.0102 (8)
C5	0.0555 (10)	0.0402 (9)	0.0516 (10)	−0.0024 (7)	0.0069 (8)	0.0044 (7)
C20	0.0602 (10)	0.0450 (9)	0.0533 (11)	−0.0006 (8)	0.0188 (9)	0.0111 (8)
C15	0.0582 (10)	0.0401 (9)	0.0515 (10)	0.0023 (7)	−0.0028 (8)	−0.0043 (8)
C2	0.0580 (10)	0.0445 (9)	0.0454 (10)	0.0083 (8)	−0.0041 (8)	−0.0043 (8)
C21	0.0518 (9)	0.0504 (10)	0.0585 (11)	−0.0133 (8)	0.0052 (8)	0.0055 (9)
C3	0.0692 (11)	0.0344 (8)	0.0580 (11)	0.0036 (8)	−0.0092 (9)	−0.0027 (8)
C4	0.0653 (11)	0.0384 (9)	0.0625 (12)	−0.0048 (8)	0.0017 (9)	0.0067 (8)
C14	0.0538 (10)	0.0511 (10)	0.0455 (10)	0.0071 (8)	0.0040 (8)	−0.0098 (8)

### Geometric parameters (Å, º)

**Table:**

S1—C1	1.7508 (16)	C12—H12	0.9300
S1—C8	1.7518 (15)	C16—C15	1.360 (2)
C11—C12	1.405 (2)	C16—H16	0.9300
C11—C13	1.415 (2)	C18—C19	1.382 (2)
C11—C10	1.430 (2)	C18—H18	0.9300
C10—C16	1.421 (2)	C19—C20	1.371 (3)
C10—C9	1.425 (2)	C19—H19	0.9300
C17—C22	1.385 (2)	C5—C4	1.372 (2)
C17—C18	1.385 (2)	C5—H5	0.9300
C17—C9	1.489 (2)	C20—C21	1.363 (3)
C7—C12	1.368 (2)	C20—H20	0.9300
C7—C8	1.421 (2)	C15—C14	1.402 (2)
C7—C6	1.450 (2)	C15—H15	0.9300
C9—C8	1.380 (2)	C2—C3	1.370 (3)
C13—C14	1.356 (2)	C2—H2	0.9300
C13—H13	0.9300	C21—H21	0.9300
C22—C21	1.383 (2)	C3—C4	1.386 (3)
C22—H22	0.9300	C3—H3	0.9300
C1—C2	1.391 (2)	C4—H4	0.9300
C1—C6	1.393 (2)	C14—H14	0.9300
C6—C5	1.392 (2)		
			
C1—S1—C8	91.35 (7)	C15—C16—C10	121.39 (15)
C12—C11—C13	121.30 (14)	C15—C16—H16	119.3
C12—C11—C10	119.76 (13)	C10—C16—H16	119.3
C13—C11—C10	118.92 (14)	C19—C18—C17	120.63 (16)
C16—C10—C9	122.76 (14)	C19—C18—H18	119.7
C16—C10—C11	117.65 (14)	C17—C18—H18	119.7
C9—C10—C11	119.57 (13)	C20—C19—C18	119.93 (17)
C22—C17—C18	118.47 (14)	C20—C19—H19	120.0
C22—C17—C9	121.64 (14)	C18—C19—H19	120.0
C18—C17—C9	119.89 (14)	C4—C5—C6	119.59 (17)
C12—C7—C8	119.28 (14)	C4—C5—H5	120.2
C12—C7—C6	128.98 (14)	C6—C5—H5	120.2
C8—C7—C6	111.74 (13)	C21—C20—C19	120.25 (16)
C8—C9—C10	118.13 (13)	C21—C20—H20	119.9
C8—C9—C17	120.69 (14)	C19—C20—H20	119.9
C10—C9—C17	121.14 (13)	C16—C15—C14	120.59 (16)
C14—C13—C11	121.33 (16)	C16—C15—H15	119.7
C14—C13—H13	119.3	C14—C15—H15	119.7
C11—C13—H13	119.3	C3—C2—C1	118.14 (17)
C9—C8—C7	122.47 (14)	C3—C2—H2	120.9
C9—C8—S1	125.69 (12)	C1—C2—H2	120.9
C7—C8—S1	111.84 (11)	C20—C21—C22	120.21 (16)
C21—C22—C17	120.47 (16)	C20—C21—H21	119.9
C21—C22—H22	119.8	C22—C21—H21	119.9
C17—C22—H22	119.8	C2—C3—C4	121.47 (16)
C2—C1—C6	121.15 (15)	C2—C3—H3	119.3
C2—C1—S1	126.11 (13)	C4—C3—H3	119.3
C6—C1—S1	112.73 (12)	C5—C4—C3	120.33 (17)
C5—C6—C1	119.26 (15)	C5—C4—H4	119.8
C5—C6—C7	128.46 (15)	C3—C4—H4	119.8
C1—C6—C7	112.27 (13)	C13—C14—C15	120.07 (16)
C7—C12—C11	120.72 (14)	C13—C14—H14	120.0
C7—C12—H12	119.6	C15—C14—H14	120.0
C11—C12—H12	119.6		
			
C12—C11—C10—C16	176.46 (14)	S1—C1—C6—C5	177.03 (13)
C13—C11—C10—C16	−2.1 (2)	C2—C1—C6—C7	178.72 (14)
C12—C11—C10—C9	−2.0 (2)	S1—C1—C6—C7	−1.81 (17)
C13—C11—C10—C9	179.41 (13)	C12—C7—C6—C5	4.2 (3)
C16—C10—C9—C8	−178.29 (14)	C8—C7—C6—C5	−176.05 (16)
C11—C10—C9—C8	0.1 (2)	C12—C7—C6—C1	−177.08 (15)
C16—C10—C9—C17	−0.9 (2)	C8—C7—C6—C1	2.66 (19)
C11—C10—C9—C17	177.53 (13)	C8—C7—C12—C11	0.5 (2)
C22—C17—C9—C8	−114.77 (18)	C6—C7—C12—C11	−179.82 (15)
C18—C17—C9—C8	66.1 (2)	C13—C11—C12—C7	−179.73 (14)
C22—C17—C9—C10	67.9 (2)	C10—C11—C12—C7	1.7 (2)
C18—C17—C9—C10	−111.22 (17)	C9—C10—C16—C15	179.23 (15)
C12—C11—C13—C14	−176.64 (16)	C11—C10—C16—C15	0.8 (2)
C10—C11—C13—C14	1.9 (2)	C22—C17—C18—C19	−2.0 (2)
C10—C9—C8—C7	2.1 (2)	C9—C17—C18—C19	177.19 (16)
C17—C9—C8—C7	−175.29 (14)	C17—C18—C19—C20	0.9 (3)
C10—C9—C8—S1	−177.76 (11)	C1—C6—C5—C4	0.3 (3)
C17—C9—C8—S1	4.8 (2)	C7—C6—C5—C4	178.93 (16)
C12—C7—C8—C9	−2.5 (2)	C18—C19—C20—C21	1.1 (3)
C6—C7—C8—C9	177.76 (14)	C10—C16—C15—C14	0.8 (3)
C12—C7—C8—S1	177.44 (12)	C6—C1—C2—C3	2.6 (2)
C6—C7—C8—S1	−2.33 (16)	S1—C1—C2—C3	−176.83 (13)
C1—S1—C8—C9	−178.97 (14)	C19—C20—C21—C22	−2.1 (3)
C1—S1—C8—C7	1.12 (12)	C17—C22—C21—C20	1.0 (3)
C18—C17—C22—C21	1.0 (3)	C1—C2—C3—C4	−0.6 (3)
C9—C17—C22—C21	−178.11 (16)	C6—C5—C4—C3	1.6 (3)
C8—S1—C1—C2	179.84 (15)	C2—C3—C4—C5	−1.5 (3)
C8—S1—C1—C6	0.41 (12)	C11—C13—C14—C15	−0.3 (3)
C2—C1—C6—C5	−2.4 (2)	C16—C15—C14—C13	−1.1 (3)

### Hydrogen-bond geometry (Å, º)

Cg2 and Cg3 are the centroids of the C1-C6 and C10–C16 rings, respectively.

**Table:**

*D*—H···*A*	*D*—H	H···*A*	*D*···*A*	*D*—H···*A*
C5—H5···*Cg*2^i^	0.93	2.94	3.8138 (19)	158
C13—H13···*Cg*3^i^	0.93	2.64	3.5399 (17)	163

Symmetry code: (i) −*x*−1/2, *y*, *z*−1/2.

## References

[bb1] Bruker (2008). *APEX2* and *SAINT* Bruker AXS Inc., Madison Wisconsin, USA.

[bb2] Farrugia, L. J. (2012). *J. Appl. Cryst.* **45**, 849–854.

[bb3] Isloora, A. M., Kalluraya, B. & Sridhar Pai, K. (2010). *Eur. J. Med. Chem.* **45**, 825–830. 10.1016/j.ejmech.2009.11.015 19945198

[bb4] Sheldrick, G. M. (2008). *Acta Cryst.* A**64**, 112–122. 10.1107/S0108767307043930 18156677

[bb5] Spek, A. L. (2009). *Acta Cryst.* D**65**, 148–155. 10.1107/S090744490804362X PMC2631630 19171970

